# Clinical and epidemiologic characteristics associated with dengue during and outside the 2016 outbreak identified in health facility-based surveillance in Ouagadougou, Burkina Faso

**DOI:** 10.1371/journal.pntd.0007882

**Published:** 2019-12-06

**Authors:** Jacqueline K. Lim, Yaro Seydou, Mabel Carabali, Ahmed Barro, Desire Lucien Dahourou, Kang Sung Lee, Teguewende Nikiema, Suk Namkung, Jung-Seok Lee, Mee Young Shin, Emmanuel Bonnet, Therese Kagone, Losseni Kaba, Tansy Edwards, Paul-André Somé, Jae Seung Yang, Neal Alexander, In-Kyu Yoon, Valéry Ridde

**Affiliations:** 1 Global Dengue and *Aedes*-transmitted diseases Consortium, International Vaccine Institute, Seoul, Korea; 2 Faculty of Epidemiology and Population Health, London School of Hygiene and Tropical Medicine, London, United Kingdom; 3 Department of public health epidemiology, Centre MURAZ, Bobo-Dioulasso, Burkina Faso, Africa; 4 Department of Epidemiology, Biostatistics, and Occupational Health, McGill University, Montreal, Quebec, Canada; 5 Action-Gouvernance-Integration-Renforcement (AGIR), Program Equité, Ouagadougou, Burkina Faso, Africa; 6 Institut de Recherché en Sciences de la Santé, Ouagadougou, Burkina Faso, Africa; 7 Department Of Zoology, University of Oxford, Oxford, United Kingdom; 8 Institute for Research on Sustainable Development (IRD), UMI Résilience, Bondy, France; 9 Centre National de Transfusion Sanguine, Ouagadougou, Burkina Faso, Africa; 10 Institute for Research on Sustainable Development (IRD), CEPED, Université de Paris, ERL INSERM SAGESUD, Paris, France; DoD - AFHSB, UNITED STATES

## Abstract

**Background:**

In Africa, the magnitude of dengue virus (DENV) transmission is largely unknown. In Burkina Faso, several outbreaks have been reported and data are often based on findings from outbreak investigations.

**Methods:**

To better understand dengue epidemiology and clinical characteristics in Burkina Faso, a fever surveillance study was conducted among patients aged 1–55 years, who presented with non-malarial febrile illness at five primary healthcare facilities in Ouagadougou, Burkina Faso from December 2014 to February 2017, encompassing a 3-month dengue outbreak in September-November 2016. Acute and convalescent blood samples were collected within an interval of 10–21 days between visits. Acute samples were tested with dengue rapid diagnostic tests (RDT) and a selected subset with RT-PCR, and all acute/convalescent samples with IgM/IgG ELISA.

**Results:**

Among 2929 non-malarial febrile patients, 740 (25%) were dengue–positive based on RT-PCR and/or IgM/IgG ELISA; 428 out of 777 patients (55%) and 312 out of 2152 (14%) were dengue-positive during outbreak and non-outbreak periods, respectively. There were 11% (316/2929) and 4% (129/2929) patients showing positive for NS1 and IgM, on the RDT, respectively. DENV 2 predominated during the outbreak, whereas DENV 3 predominated before the outbreak. Only 25% of dengue-positive cases were clinically diagnosed with suspected dengue. The odds of requiring observation for ≤3 days (versus routine outpatient care) were 11 times higher among dengue-positive cases than non-dengue cases. In adjusted analyses, dengue-positivity was associated with rash and retro-orbital pain (OR = 2.6 and 7.4, respectively) during the outbreak and with rash and nausea/vomiting (OR = 1.5 and 1.4, respectively) during the non-outbreak period.

**Conclusion:**

Dengue virus is an important pathogen in Burkina Faso, accounting for a substantial proportion of non-malarial fevers both during and outside outbreak, but is only infrequently suspected by clinicians. Additional longitudinal data would help to further define characteristics of dengue for improved case detection and surveillance.

## Author summary

There is not much evidence on dengue in Africa, relative to the Asia-Pacific and Latin American regions. To estimate the proportion of dengue among patients with fever, and to identify clinical features of dengue during outbreak and non-outbreak periods, we studied 2929 patients with non-malarial fever, aged 1–55 years, who attended five primary healthcare centers in Ouagadougou, Burkina Faso. Patients were tested with a rapid test for dengue, and further tests were carried out on paired blood samples taken 10–21 days apart. Overall, a quarter of non-malarial febrile episodes identified between December 2014 and February 2017 were dengue-positive. Dengue-positive cases were 11 times more likely than non-dengue cases to require observation for ≤3 days. During the study period in 2016, there was a dengue outbreak where more than half of non-malarial febrile patients were identified to be dengue-positive. DENV 2 was the main serotype in circulation during the outbreak, whereas DENV 3 was the main serotype before the outbreak. Rash and retro-orbital pain were more frequently found among dengue-positive cases, compared to non-dengue cases, during the outbreak. During the non-outbreak period, rash and nausea/vomiting were more likely in dengue-positive versus non-dengue cases. There was a low level of clinical suspicion of dengue even during the 2016 outbreak. Therefore, a broader use of rapid diagnostic tests and more epidemiologic data would help to improve dengue case detection and surveillance in Burkina Faso.

## Introduction

Dengue Fever (DF) is a mosquito-borne disease caused by four related but antigenically distinct dengue viruses (DENVs, serotypes 1–4). Approximately 50 to 100 million cases of DF and 500,000 severe dengue cases requiring hospitalization reportedly occur annually worldwide [1–3].

The *Aedes* mosquito vectors of DENV are widely distributed in Africa, and dengue cases have been reported in 34 African countries [4–6]. However, data are limited to retrospective testing of existing samples or outbreak investigations from a few countries [5, 7–9]. Several studies have identified DENV as a common cause of febrile illness in Africa, but there is a continued challenge to distinguish dengue from other causes of febrile illness given limited diagnostic capabilities [10–12].

In Burkina Faso, several outbreaks have been reported since 1925 [5, 13, 14], including an outbreak declared in November 2013 by the Burkina Faso Ministry of Health (MoH) [11, 15]. Between 5 August and 12 November 2016, the Burkina Faso MoH conducted an outbreak investigation as part of emergency response in collaboration with World Health Organization (WHO) and 1266 suspected dengue cases were identified by the MoH, with 1061 cases positive by dengue rapid diagnostic test (RDT), and 15 deaths from all 12 districts of Ouagadougou [16, 17]. Most recently, an even larger outbreak occurred in September 2017, with 9029 suspected dengue cases, 5773 dengue RDT-positive cases, and 18 deaths throughout the country [18]. These repeated outbreaks suggest a considerable dengue burden in Burkina Faso.

Most African countries lack mandatory reporting or national surveillance systems for dengue [19]. Burkina Faso added dengue to its routine national surveillance system for diseases with epidemic-potential in 2016. Also, the MoH conducts outbreak investigations at several sentinel health centers [11].

To better understand the dengue problem in Burkina Faso, a passive facility-based fever surveillance study was conducted in Ouagadougou, from 2014–2017. During the study period, the 2016 dengue outbreak occurred, allowing for characterization of dengue epidemiology and comparison of clinical features during and outside the outbreak.

## Methods

### Study area and population

The study area was selected based on the existence of previous outbreaks and case reports, past seroprevalence and modelling studies, as well as the availability of research infrastructure [4, 20, 21]. Ouagadougou is the capital city of Burkina Faso in West Africa with most of its population residing in urban settings [22]. In March-May, temperatures may reach 43°C, and it is followed by the rainy season in May-September. Health services in Ouagadougou are provided by three university hospitals, five district hospitals, and 60 primary healthcare centers (CSPS, Centres de Santé et de Promotion Sociale), as well as private clinics [23].

**Fig 1:**
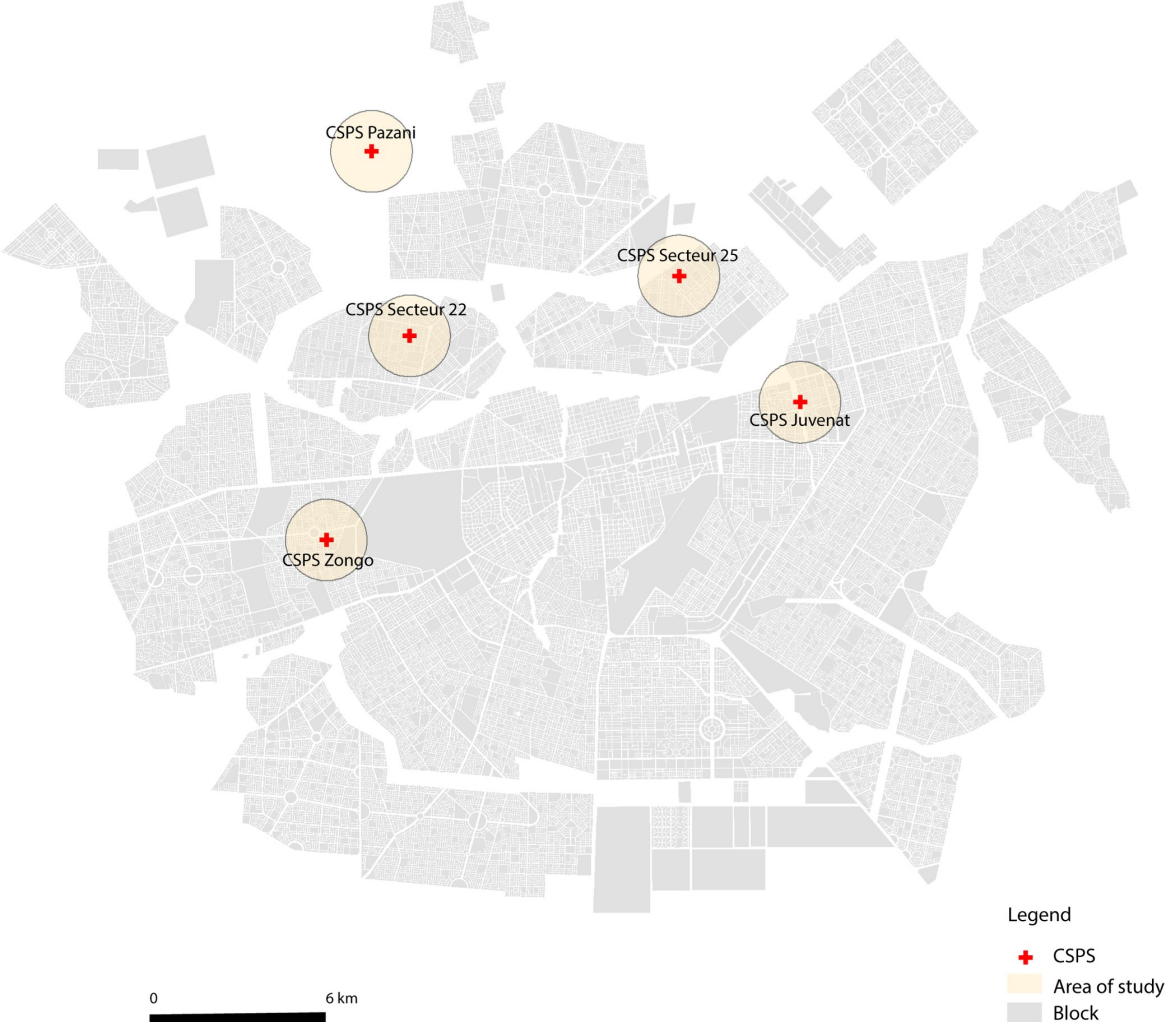
A map of the study area in Ouagadougou, Burkina Faso. The map shows the approximate location of the selected facilities of 5 CSPSs (Pazani, CSPS22, CSPS25, Juvenat Fille, Zongo), serving a catchment population of 110,000 residents of Ouagadougou, Burkina Faso [20].

The current study was implemented in five CSPSs (Pazani, CSPS22, CSPS25, Juvenat Fille, Zongo), serving a catchment population of 110,000 residents (Fig 1). The population in Ouagadougou is stable with an annual transmigration rate of 4.1% and >80% with home ownership [24].

### Study design

Investigational methods can be found in previous publication [20]. The passive facility-based fever surveillance study enrolled outpatients and observation patients (for ≤3 days), as previously described [20], between December 2014 and February 2017 (27 months). Patients presenting with fever (body temperature ≥ 37.5º C) or history of (self-reported) fever for ≤7 days were tested for malaria using RDT (SD BIOLINE Malaria kit, Standard Diagnostics, Yongin-Si, Korea) as part of routine practice. Patients were eligible for study enrolment if they were malaria RDT-negative without localizing signs (i.e., no localized infection or known/confirmed non-dengue etiology), aged 1–55 years, resident of the catchment area covered by the study CSPSs, and provided informed consent, plus assent for individuals aged 8–17 years.

**Fig 2:**
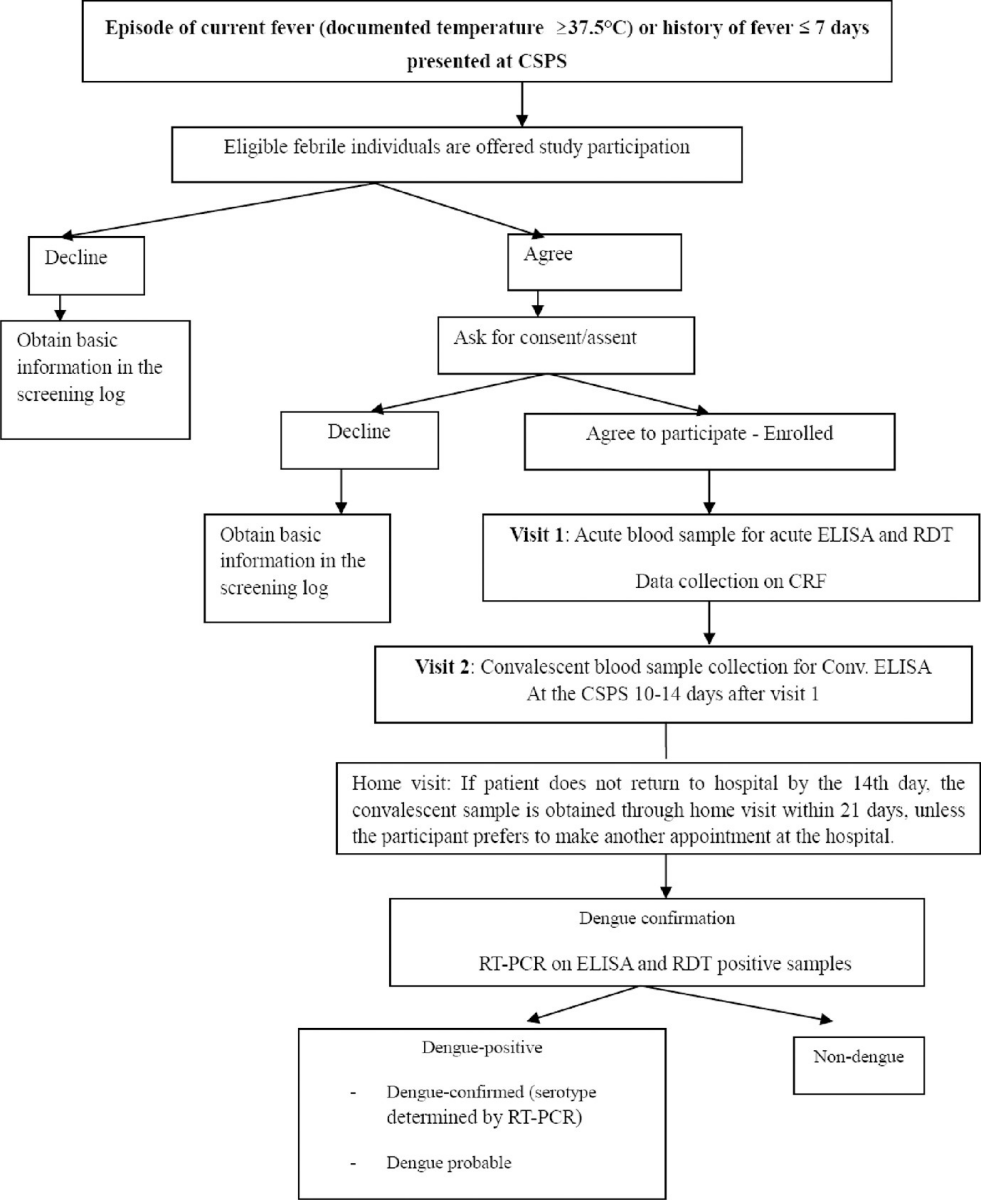
A chart of patient flow in passive fever surveillance. A chart of patient flow in passive fever surveillance- The chart shows the study flow when a febrile patient presents at the CSPS from screening, enrollment, and lab testing.

Malaria RDT-negative patients were tested using dengue RDTs. During the enrollment visit, an acute blood sample (7–10 ml) was collected (Fig 2). Then, a study physician/nurse conducted interviews and physical exams, and a surveillance case report form was completed capturing symptom history, medical history, treatment and laboratory results [20]. A convalescent blood sample was collected at the facility between 10–14 days after the initial visit, or if not possible within this timeframe, the patient was followed up at home within 21 days.

### Laboratory testing algorithm

Laboratory testing algorithm has been described in previous publication [20]. As described previously [20], acute samples were tested at enrollment at the CSPS using a commercial RDT for dengue nonstructural protein 1 (NS1) and Immunoglobulin type M and type G (IgM/IgG) (Dengue Duo, Standard Diagnostics, Yongin-Si, Korea). The acute and convalescent samples were transported in 4ºC ice boxes to Virology laboratory of CHU YO (University Hospital Center Yalgado Ouédraogo, in French: “Centre Hospitalier Universtaire Yalgado Ouédraogo”) where blood samples were centrifuged and separated into cryotubes in 0.5–1 ml serum aliquots under sterile conditions, labeled and stored at -70°C freezer. Subsequently, they were brought and tested in the Centre Muraz laboratory using dengue IgM/IgG Enzyme-Linked Immunosorbent Assay (ELISA) (SD Dengue IgM & IgG Capture ELISA, Standard Diagnostics, Yongin-Si, Korea). Furthermore, as described in previous publication [20], Reverse Transcriptase-Polymerase Chain Reaction (RT-PCR) for laboratory confirmation of dengue infection and serotyping [25] was performed at the International Vaccine Institute (IVI), on acute sera from patients who had: (i) NS1 or IgM positive by RDT in the acute sample; and/or (ii) sero-conversion between acute and convalescent samples by IgM and IgG capture ELISA. RT-PCR was also performed on a limited number of randomly selected acute sera that were: (iii) sero-positive in both acute and convalescent samples by IgM and IgG capture ELISA; or (iv) IgG positive by RDT in the acute sample; or (v) negative by RDT and ELISA on all samples.

Dengue infection status was categorized based on interpretation of laboratory results, following WHO diagnostic criteria [26]. Sero-conversion by dengue IgM and/or IgG between acute and convalescent samples and/or virus detection by RT-PCR in the acute sample were considered to be laboratory-confirmed dengue. Positive IgM by ELISA in a single acute sample or paired acute/convalescent samples, or NS1 and/or IgM positive by RDT were considered as probable dengue [26]. Confirmed and probable dengue cases were combined into a dengue-positive group for this analysis. Patients with negative RT-PCR and negative paired acute/convalescent IgM ELISA were classified as non-dengue.

### Statistical analysis

There were 2 components in the analysis. First, a descriptive summary of clinical and laboratory characteristics is presented for dengue-positive and non-dengue cases. Elevated body temperature, as a dichotomous variable, was defined as body temperature ≥38.5°C, the 75th percentile of the body temperature measured at enrollment. Clinical diagnosis (i.e., made by clinician prior to laboratory confirmation) was grouped as suspected dengue, undifferentiated fever, and other illness. Our surveillance covered the entire outbreak from September to November 2016. Cases were also designated as outbreak or non-outbreak depending on date of occurrence, with outbreak cases considered as those occurring between September and November 2016, defined to be consistent with the outbreak period declared by Burkina Faso MoH/WHO [16, 17]. Yellow fever (YF) vaccination history was dichotomized between those who reported having been vaccinated versus those who did not remember or reported no vaccination. Categorical pair-wise comparisons were made across dengue infection status using χ2 or Fisher’s exact tests with significance level of 0.05 [27]. Continuous variables were compared using Student’s t-test or ANOVA [28].

Secondly, based on our a priori hypothesis that clinical presentation associated with dengue-positivity would be different between the outbreak and non-outbreak periods, logistic regression was used to build a multivariable model of clinical indicators associated with dengue-positive vs. non-dengue cases, to separately fit the outbreak and non-outbreak periods. The models contained age and gender as a priori confounders, possibly associated with exposure to *Aedes* vectors, and with some clinical features [29]. A backward stepwise process was used to select a final multivariable model for each outbreak status, with a significance level of 0.2 for entry and 0.1 for retention. Further variables investigated included: demographic and clinical variables such as YF vaccination history, requirement for observation, fever duration prior to enrollment, temperature at presentation, and clinical signs/symptoms. Some signs and symptoms were used only in the descriptive and univariate analyses, due to data sparsity. Clinical diagnosis of suspected dengue was considered to be closely related to the outcome of dengue-positivity and was not included.

Finally, a single set of variables was obtained as the union of the sets of variables from regression modelling in the outbreak and non-outbreak periods. Variables found to be significant in only one period were applied to both periods, producing a single list of variables. These variables were fitted to both outbreak and non-outbreak periods to give comparable results between them.

As part of sensitivity analysis, a descriptive summary of clinical and laboratory characteristics using three categories for dengue infection status—confirmed, probable, and non-dengue—is presented in supplementary S2 Table. Between dengue-confirmed and non-dengue groups, univariate logistic analyses were conducted for during and outside the outbreak (S3 and S4 tables). All analyses were performed using SAS version 9.4 (SAS Institute, Cary, North Carolina).

### Ethical considerations

The study protocol received ethical approvals from the Institutional Review Boards (IRBs) of IVI (No. 2014–008), the London School of Hygiene and Tropical Medicine (Reference number: 17096), the National Ethical Committee for Health Research of Burkina Faso, and the Ethics Committee of the Centre Hospitalier de l'Université de Montréal (CRCHUM) at University of Montreal.

A written informed consent from (ICF) was obtained from each participant. For those aged between 8 and 17 years, an assent form was obtained, plus informed consent from at least one parent or legal guardian.

## Results

**Fig 3:**
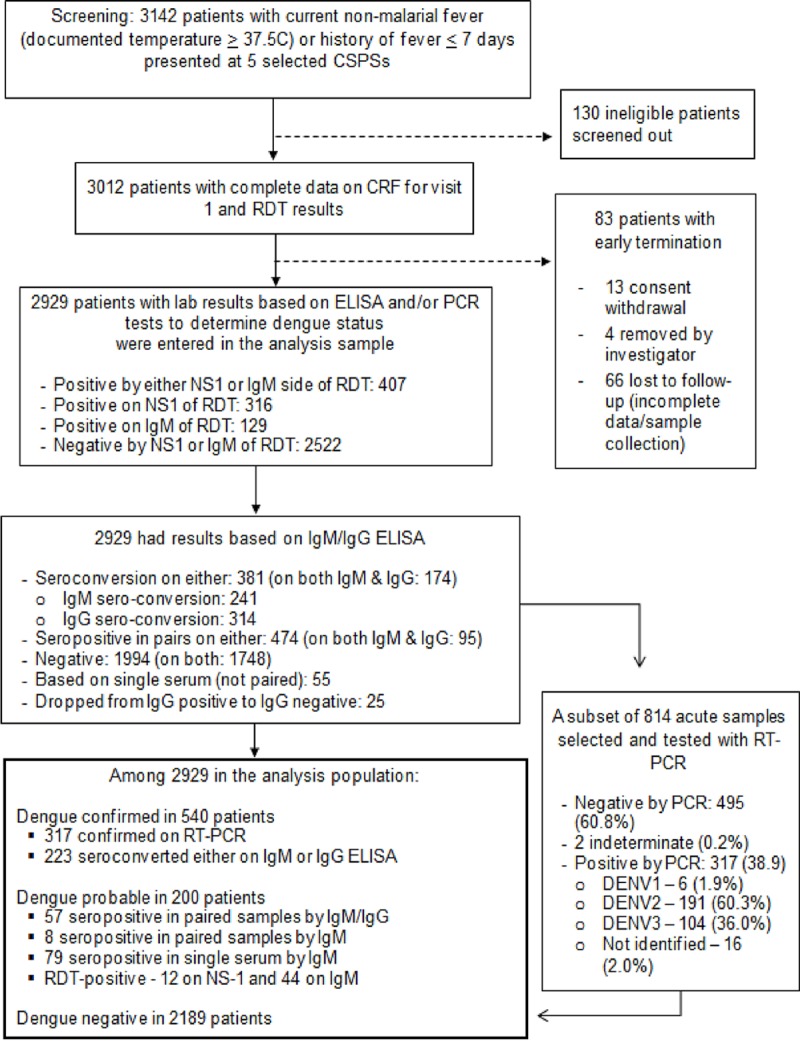
A chart of patient flow in passive fever surveillance. The diagram shows how we reached the study population and the test results from collected samples, within the surveillance.

Analysis was performed on 2929 out of 3012 enrolled patients with complete clinical and laboratory data; 83 withdrew consent or had incomplete laboratory data to determine dengue infection status (Fig 3). Although similar in terms of age, gender, requirement for observation, and days of illness before enrollment, these 83 patients were significantly different from the analysis sample in terms of residential neighborhood—the majority from Zongo (40%) and Pazani (28%)—and being mostly from non-outbreak periods (87%). In terms of missing data, only the patients requiring observation had information on the complete blood count (CBC) test and the results from CBC were not included in the analysis.

### Clinical characteristics between dengue-positive and non-dengue cases

**Fig 4:**
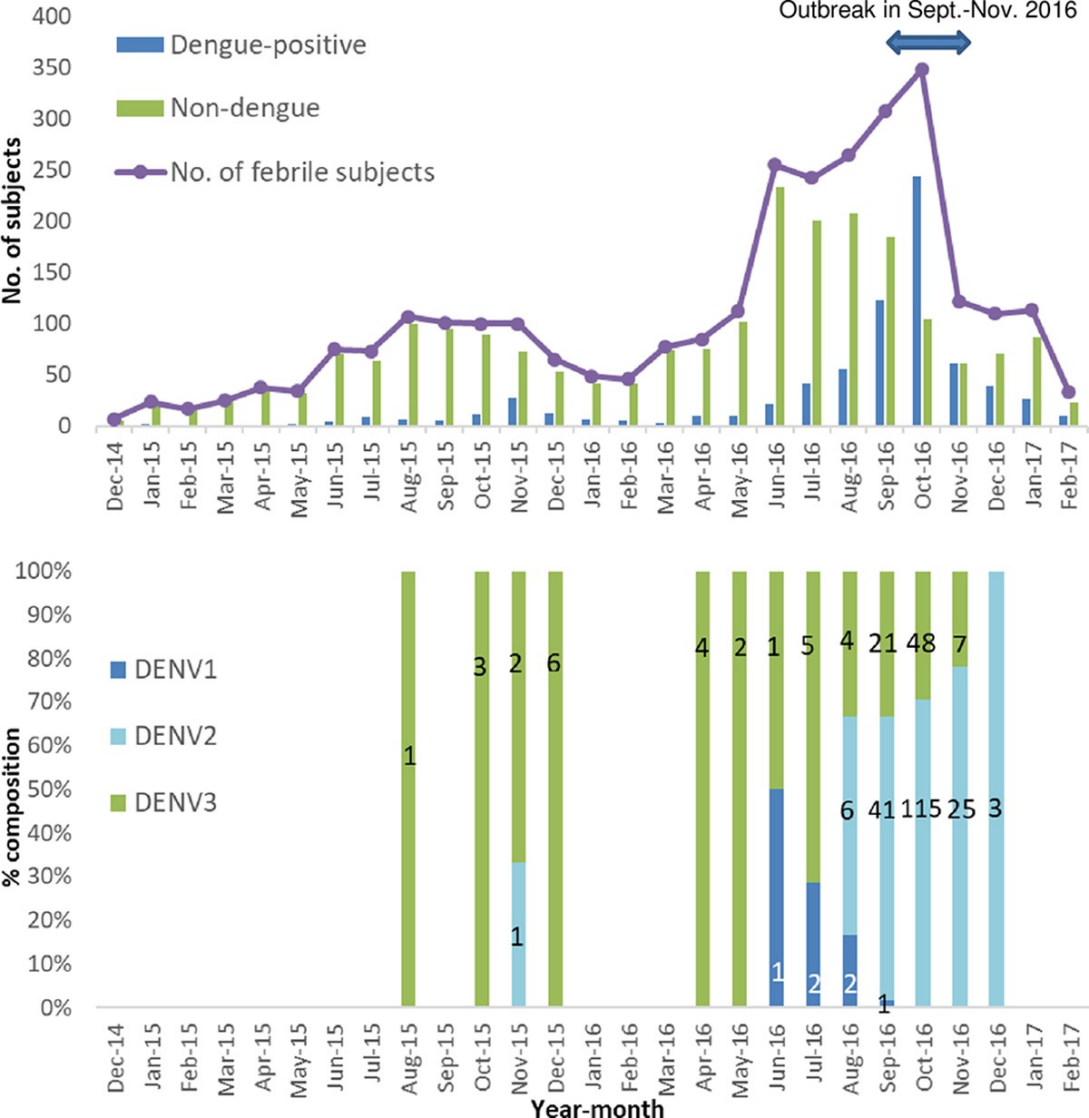
Monthly distribution of febrile enrollees, dengue-positive and non-dengue cases & monthly distribution of dengue serotypes* in PCR-positive cases. The figure has two parts: the upper part shows monthly distribution of dengue-positive and non-dengue cases among the enrolled patients; and the lower part shows distribution of serotypes identified (numbers shown in the bars) by month. *number of identified serotypes shown in the bars.

Table 1 describes demographic and clinical characteristics of dengue-positive vs. non-dengue cases. Of 2929 analyzed patients, 2189 (74.7%) were non-dengue and 740 (25.3%) were dengue-positive. Of the 740 dengue-positive patients, 540 (73.0%) were laboratory-confirmed and 200 (27.0%) were probable dengue. Of the dengue-positive cases, 42% (n = 317) were confirmed by RT-PCR and the remainder by paired ELISA (Fig 3). A small peak in dengue-positive cases was observed in October-December 2015. A much larger peak occurred in August-December 2016 (Fig 4). Both peaks occurred at the end or after the May-September rainy season. Of 777 fever cases from the outbreak, 55.1% (n = 428) were dengue-positive, with DENV2 predominating [181/258 (70%) of samples confirmed by RT-PCR] (Fig 4). Of 2152 non-outbreak fever cases, 14.5% (n = 312) were dengue-positive, mostly with DENV3 [28/43 (65%) of samples confirmed by RT-PCR] and a few DENV1 [5/43 (12%) of samples confirmed by RT-PCR].

**Table 1:** Demographic and clinical characteristics of dengue-positive and non-dengue cases in the facility-based fever surveillance established in Ouagadougou, Burkina Faso, between December 2014 and February 2017.

Characteristics	Dengue-positive (n = 740)	Non-dengue (n = 2189)	Total (n = 2929)	p-value
Age group (years)				**< .001**
1–4	37 (5.0)	275 (12.6)	312 (10.7)	
5–9	43 (5.8)	149 (6.8)	192 (6.6)	
10–14	45 (6.1)	129 (5.9)	174 (5.9)	
15–19	85 (11.5)	231 (10.6)	316 (10.8)	
20–24	110 (14.9)	366 (16.7)	476 (16.3)	
25–29	134 (18.1)	375 (17.1)	509 (17.4)	
30–34	94 (12.7)	269 (12.3)	363 (12.4)	
35–39	71 (9.6)	155 (7.1)	226 (7.7)	
40–44	57 (7.7)	111 (5.1)	168 (5.7)	
45–49	33 (4.5)	67 (3.1)	100 (3.4)	
50–55	31 (4.2)	62 (2.8)	93 (3.2)	
Female	465 (62.8)	1563 (71.4)	2028 (69.2)	**< .001**
CSPS				**< .001**
Pazani	113 (15.3)	400 (18.3)	513 (17.5)	
Zongo	91 (12.3)	592 (27.0)	683 (23.3)	
CSPS22	65 (8.8)	240 (11.0)	305 (10.4)	
CSPS25	266 (36.0)	502 (22.9)	768 (26.2)	
Juvenat Fille	205 (27.7)	446 (20.4)	651 (22.2)	
Under observation ≤3 days/OPD	135 (18.2)/605 (81.8)	45 (2.1)/2144 (97.9)	180 (6.2)/2749 (93.9)	**< .001**
Mean days, fever duration prior to visit (SD)	2.92 (1.21)	2.61 (1.22)	2.69 (1.23)	**< .001**
Fever duration prior to visit				**< .001**
1–2 days	301 (40.7)	1153 (52.7)	1454 (49.6)	
3 days	238 (32.2)	634 (29.0)	872 (29.8)	
4–7 days	201 (27.2)	400 (18.4)	603 (20.6)	
Mean temperature at enrollment (SD)	38.29 (0.77)	38.03 (0.78)	38.09 (0.78)	**< .001**
Temperature at enrollment				**< .001**
Below 38.5°c	478 (64.6)	1681 (76.8)	2159 (73.7)	
≥ 38.5°c	262 (35.4)	508 (23.2)	770 (26.3)	
Mean days, fever duration, entire illness (SD)	4.72 (2.52)	4.04 (2.46)	4.21 (2.49)	**< .001**
Prev. dengue infection (self-report)	14 (1.9)	2 (0.1)	16 (0.6)	**< .001**
YF vaccination (self-report)				**< .001**
Received	122 (16.5)	824 (37.6)	946 (32.3)	
Not received	618 (83.5)	1365 (62.4)	1983 (67.7)	
Clinical diagnosis				
Suspected dengue	187 (25.3)	12 (0.6)	199 (6.8)	**< .001**
Undifferentiated fever	529 (71.5)	1987 (90.8)	2516 (85.9)	
Other illness	24 (3.2)	190 (8.7)	214 (7.3)	
URI (% of other illness)	5 (20.8)	27 (14.2)	32 (15.0)	
Bronchitis	4 (16.7)	30 (15.8)	34 (15.9)	
Pneumonia	6 (25.0)	21 (11.1)	27 (12.6)	
Viral syndrome	3 (12.5)	11 (5.8)	14 (6.5)	
Diarrheal illness	2 (8.3)	28 (14.7)	30 (14.0)	
Influenza	1 (4.2)	4 (2.1)	5 (2.3)	
Others	3 (12.5)	69 (36.3)	72 (33.6)	

**Fig 5:**
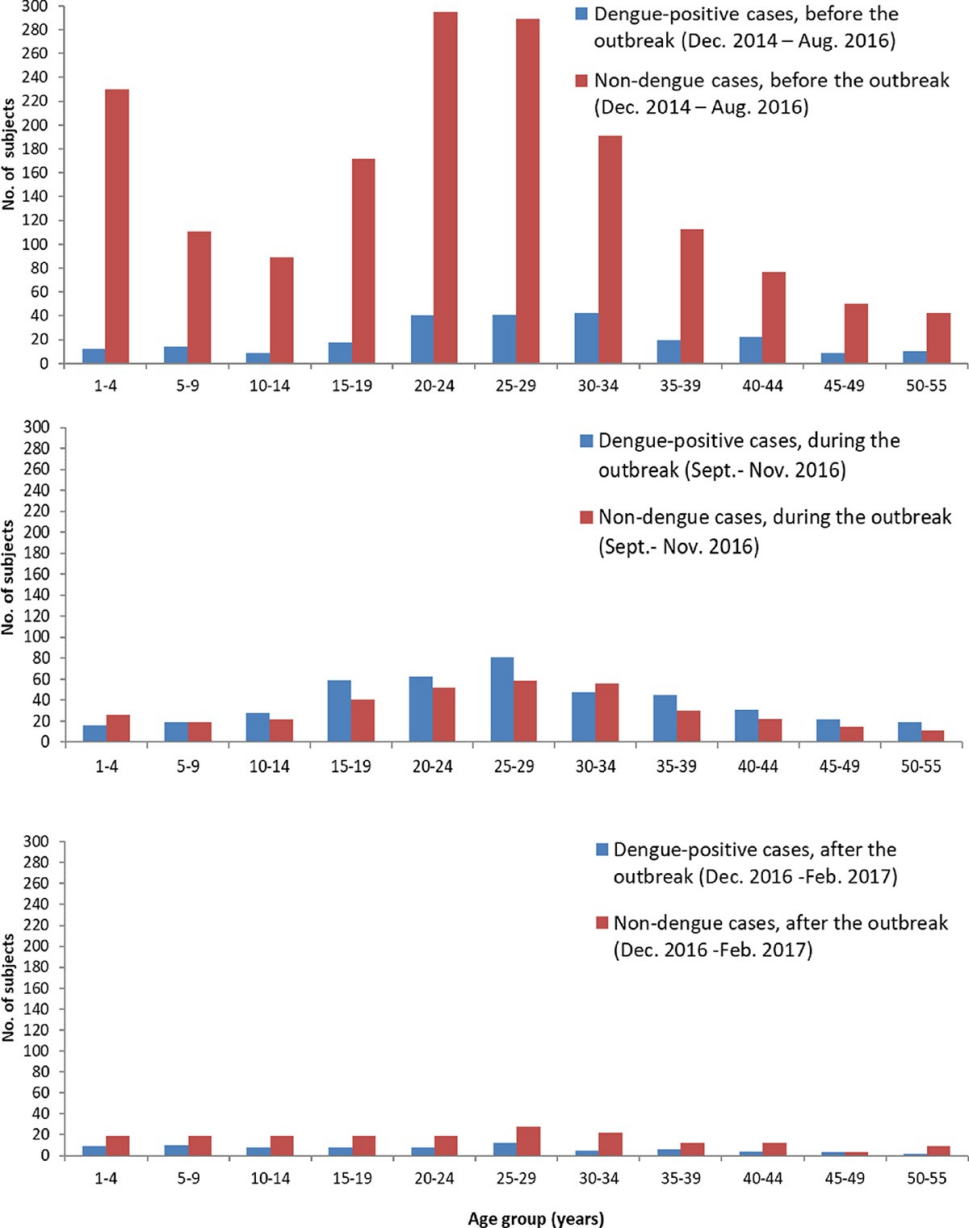
Age distribution of dengue-positive cases before, during, and after the 2016 outbreak. The figure shows age distribution of dengue-positive cases, compared to non-dengue cases, before, during, and after the 2016 outbreak.

Overall, dengue-positive cases were older than non-dengue cases (Table 1). Among dengue-positive cases, those after the 2016 outbreak were younger than those before or during the outbreak (about 75% <30 years old, compared to before and during the outbreak with about 50% <30 years) (Fig 5); the age difference before, during and after the outbreak was statistically significant (ANOVA, p-value < .001). Differences in terms of presenting signs and symptoms are presented in Table 2.

**Table 2:** Signs and symptoms of dengue-positive and non-dengue cases in the facility-based fever surveillance established in Ouagadougou, Burkina Faso, between December 2014 and February 2017.

Presence of signs and symptoms	Dengue-positive (n = 740)	Non-dengue (n = 2189)	Total (n = 2929)	p-value
Rash	95 (12.8)	163 (7.5)	258 (8.8)	**< .001**
Fatigue	603 (81.5)	1526 (69.7)	2129 (72.7)	**< .001**
Headache	708 (95.7)	1899 (86.8)	2607 (89.0)	**< .001**
Retro-orbital pain	131 (17.7)	107 (4.9)	238 (8.1)	**< .001**
Neck pain	13 (1.8)	47 (2.2)	60 (2.1)	0.517
Ear pain	2 (0.3)	10 (0.5)	12 (0.4)	0.741
Nasal congestion	20 (2.7)	105 (4.8)	125 (4.3)	**0.015**
Rhinorrhea	30 (4.1)	132 (6.0)	162 (5.5)	**0.042**
Sore Throat	11 (1.5)	64 (2.9)	75 (2.6)	**0.032**
Cough	91 (12.3)	354 (16.2)	445 (15.2)	**0.011**
Sputum production	4 (0.5)	30 (1.4)	34 (1.2)	0.075
Nausea & vomiting	270 (36.5)	635 (29.0)	905 (30.9)	**< .001**
Diarrhea	23 (3.1)	128 (5.9)	151 (5.2)	**0.004**
Constipation	12 (1.6)	85 (3.9)	97 (3.3)	**0.003**
Abdominal pain	271 (36.6)	639 (29.2)	910 (31.1)	**< .001**
Nose bleeding	7 (1.0)	10 (0.5)	17 (0.6)	0.130
Gum bleeding	5 (0.7)	2 (0.1)	7 (0.2)	0.013
Loss of appetite	331 (44.7)	739 (33.8)	1070 (36.5)	**< .001**
Capillary refill >2 sec	8 (1.1)	19 (0.9)	27 (0.9)	0.600
Myalgia	319 (43.1)	560 (25.6)	879 (30.0)	**< .001**
Arthralgia	426 (57.6)	953 (43.5)	1379 (47.1)	**< .001**

There were 180 patients requiring observation at the CSPS. Patients later determined to be dengue-positive were more likely, on presentation, to require observation: 18% of dengue-positive cases versus 2% of non-dengue cases (Table 1). A small but significant difference was observed in average time between fever onset and enrollment for dengue-positive versus non-dengue cases (2.9 days vs. 2.6 days, p < .001). Likewise, the entire duration of fever illness on average was significantly longer for dengue-positive cases (mean 4.7 versus 4.0 days, among the 2926 patients with such data, p < .001). Dengue-positive cases were half as likely to self-report that they had been vaccinated for YF (17%, versus 38% for non-dengue cases, p < .001).

Of 2929 available RDT results, 11% (316/2929) and 4% (129/2929) were positive for NS1 and IgM, on the RDT kit, respectively (Fig 1). There were 38 patients [28 (74%) during the outbreak and 10 (26%) during the non-outbreak periods] with positive results for both NS1 and IgM on the RDT. During the outbreak period, 86% (271/316) were NS-1 positive and 40% (52/129) were IgM positive (28 showing positive on both NS1 and IgM).

Only 25% of dengue-positive cases were clinically diagnosed with suspected dengue, prior to lab-confirmation, and more than 90% of non-dengue cases were clinically diagnosed with undifferentiated fever. During the outbreak, 31.3% (131/428) of dengue-positive cases were diagnosed with suspected dengue, while 17.0% (53/312) were diagnosed with suspected dengue during non-outbreak periods.

### Clinical features associated with dengue during and outside the 2016 outbreak

Over the outbreak period, of the 740 dengue-positive patients, 357 patients (48%) were laboratory-confirmed. Of these dengue-confirmed cases, 258 (72%) were confirmed by RT-PCR; 55 (15%) by both IgM and IgG seroconversion; and 44 (12%) by IgM or IgG seroconversion on paired ELISA. Over the non-outbreak period, of the 740 dengue-positive patients, 183 (25%) patients were laboratory-confirmed. Of these dengue-confirmed cases, 59 (32%) were confirmed by RT-PCR; 10 (5%) by both IgM and IgG seroconversion; and 114 (62%) by IgM or IgG seroconversion.

Demographic and clinical associations with dengue-positivity are shown in Table 3 for the outbreak and in Table 4 for non-outbreak periods. During the outbreak, independently associated symptoms were: rash, retro-orbital pain, cough, headache, nausea/vomiting, and loss of appetite. During non-outbreak periods, retro-orbital pain, headache, nausea/vomiting, and constipation were independently associated. In addition to the symptoms, the multivariable model selected requirement for observation and lack of YF vaccination to be associated with dengue-positivity in both outbreak and non-outbreak periods. Age in non-outbreak periods and, gender, elevated temperature at enrollment, and fever duration prior to enrollment in the outbreak period were also selected. Age and gender were a priori confounders and were significantly associated with dengue-positivity. Enrolled CSPS may be a proxy for otherwise any unexplained variation across centers, but was not selected for either of the outbreak or non-outbreak periods. In the absence of observation of variation with respect to dengue-positivity, it was not entered in the models.

**Table 3:** Univariate logistic analyses showing significant indicators and their odds ratios of dengue-positivity during the outbreak period, from the facility-based fever surveillance established in Ouagadougou, Burkina Faso, between December 2014 and February 2017.

Characteristics	During outbreak (n = 777)					
	Total N	N (%) dengue-positive (n = 428)	N (%) Non- dengue (n = 349)	Univariate analysis Dengue-positive vs. non-dengue		
				OR	95% Confidence Interval (CI)	p-Value
Age group (years)						0.195
1–14	129	63 (48.8)	66 (51.2)	Ref	-	
15–24	213	121 (56.8)	92 (43.2)	1.38	0.89–2.14	
25–34	242	128 (52.9)	114 (47.1)	1.18	0.77–1.80	
35–55	193	116 (60.1)	77 (39.9)	**1.58**	**1.01–2.47**	
Gender*						**0.004**
Male	293	181 (61.8)	112 (38.2)	Ref	-	
Female	484	247 (51.0)	237 (49.0)	**0.65**	**0.48–0.87**	
Under observation** (*ref*. OPD)	128	110 (85.9)	18 (14.1)	**6.36**	**3.77–10.71**	**< .001**
Fever duration prior to visit*						**0.007**
1–2 days	330	168 (50.9)	162 (49.1)	Ref	-	
3 days	244	129 (52.9)	115 (47.1)	1.08	0.78–1.51	
4–7 days	203	131 (64.5)	72 (35.5)	**1.75**	**1.23–2.51**	
Temperature at enrollment*						**0.009**
Below 38.5°c	468	240 (51.3)	228 (48.7)	**Ref**	**-**	
≥ 38.5°c	309	188 (60.8)	121 (39.2)	**1.48**	**1.10–1.98**	
No YF vaccination†* (*ref*. received vaccination)	630	363 (57.6)	267 (42.4)	**1.72**	**1.19–2.46**	**0.004**
Presence of signs and symptoms (*ref*. absence)						
Rash*	84	60 (71.4)	24 (28.6)	**2.21**	**1.34–3.63**	**0.002**
Fatigue*	620	353 (56.9)	267 (43.1)	**1.45**	**1.02–2.05**	**0.040**
Retro-orbital pain**	104	92 (88.5)	12 (11.5)	**7.69**	**4.14–14.30**	**< .001**
Headache*	749	420 (56.1)	329 (43.9)	**3.19**	**1.39–7.33**	**0.006**
Nasal congestion*	21	5 (23.8)	16 (76.2)	**0.25**	**0.09–0.68**	**0.007**
Rhinorrhea*	28	7 (25.0)	21 (75.0)	**0.26**	**0.11–0.62**	**0.002**
Cough**	81	28 (34.6)	53 (65.4)	**0.39**	**0.24–0.63**	**< .001**
Nausea & vomiting	285	154 (54.0)	131 (46.0)	0.94	0.70–1.25	0.655
Diarrhea	21	8 (38.1)	13 (61.9)	0.49	0.20–1.20	0.120
Abdominal pain	263	153 (58.2)	110 (41.8)	1.21	0.90–1.63	0.216
Loss of appetite	383	217 (56.7)	166 (43.3)	1.13	0.85–1.50	0.385
Myalgia**	366	227 (62.0)	139 (38.0)	**1.71**	**1.28–2.27**	**< .001**
Arthralgia	521	295 (56.6)	226 (43.4)	1.21	0.89–1.63	0.219

Statistical significance of the frequencies

*p-value<0.05

**p-value < .001

†based on self-report

**Table 4:** Univariate logistic analyses showing significant indicators and their odds ratios of dengue-positivity during non-outbreak periods, from the facility-based fever surveillance established in Ouagadougou, Burkina Faso, between December 2014 and February 2017.

Characteristics	During non-outbreak (n = 2152)					
	Total N	N (%) dengue-positive (n = 312)	N (%) Non- dengue (n = 1840)	Univariate analysis Dengue-positive vs. non-dengue		
				OR	95% CI	p-Value
Age group (years)*						**0.003**
1–14	549	62 (11.3)	487 (88.7)	Ref	-	
15–24	579	74 (12.8)	505 (87.2)	1.15	0.80–1.65	
25–34	630	100 (15.9)	530 (84.1)	**1.48**	**1.06–2.08**	
35–55	394	76 (19.3)	318 (80.7)	**1.88**	**1.31–2.70**	
Gender						
Male	608	94 (15.5)	514 (84.5)	Ref	-	
Female	1544	218 (14.1)	1326 (85.9)	0.90	0.69–1.17	0.426
Under observation** (*ref*. OPD)	52	25 (48.1)	27 (51.9)	**5.85**	**3.35–10.22**	**< .001**
Fever duration prior to visit*						**0.001**
1–2 days	1124	133 (11.8)	991 (88.2)	**Ref**	**-**	
3 days	628	109 (17.4)	519 (82.6)	**1.57**	**1.19–2.06**	
4–7 days	400	70 (17.5)	330 (82.5)	**1.58**	**1.15–2.17**	
Temperature at enrollment						0.285
Below 38.5°c	1691	238 (14.1)	1453 (85.9)	Ref	-	
≥ 38.5°c	461	74 (16.1)	387 (84.0)	1.17	0.88–1.55	
No YF vaccination†** (*ref*. received vaccination)	1353	225 (18.9)	1098 (81.2)	**3.02**	**2.24–4.09**	**< .001**
Presence of signs and symptoms (*ref*. absence)						
Rash*	174	35 (20.1)	139 (79.9)	**1.55**	**1.05–2.29**	**0.029**
Fatigue**	1509	250 (16.6)	1259 (83.4)	**1.86**	**1.39–2.50**	**< .001**
Retro-orbital pain**	134	39 (29.1)	95 (70.9)	**2.62**	**1.77–3.89**	**< .001**
Headache**	1858	288 (15.5)	1570 (84.5)	**2.06**	**1.33–3.19**	**0.001**
Nasal congestion	104	15 (14.4)	89 (85.6)	0.99	0.57–1.74	0.982
Rhinorrhea	134	23 (17.2)	111 (82.8)	1.24	0.78–1.98	0.366
Cough	364	63 (17.3)	301 (82.7)	1.29	0.96–1.75	0.096
Nausea & vomiting**	620	116 (18.7)	504 (81.3)	**1.57**	**1.22–2.02**	**< .001**
Diarrhea	130	15 (11.5)	115 (88.5)	0.76	0.44–1.32	0.325
Abdominal pain*	647	118 (18.2)	529 (81.8)	**1.51**	**1.17–1.94**	**0.001**
Loss of appetite	687	114 (16.6)	573 (83.4)	1.27	0.99–1.64	0.059
Myalgia*	513	92 (29.5)	421 (82.1)	**1.41**	**1.08–1.84**	**0.012**
Arthralgia	858	131 (15.3)	727 (84.7)	1.11	0.87–1.41	0.409

Statistical significance of the frequencies

*p-value<0.05

**p-value < .001

†based on self-report

Table 5 shows the final set of variables. During both outbreak and non-outbreak periods, dengue-positive patients had increased odds of presenting with rash [outbreak: 2.6 (95%CI = 1.5–4.6); non-outbreak: 1.5 (95%CI = 1.0–2.4)] and retro-orbital pain [outbreak: 7.4 (95%CI = 3.7–14.7); non-outbreak: 1.4 (95%CI = 1.01–1.8)].

**Table 5:** Multivariate logistic analysis showing significant indicators and their odds ratios of dengue-positivity by outbreak or non-outbreak periods, in the facility-based fever surveillance established in Ouagadougou, Burkina Faso, between December 2014 and February 2017.

Characteristics	Multivariate analysis					
	During outbreak* (n = 777) *ref*. non-dengue (n = 349)			During non-outbreak (n = 2152) *ref*. non-dengue (n = 1840)		
	Dengue-positive (n = 428)		p-Value	Dengue-positive (n = 312)		p-Value
	aOR	95% CI		aOR	95% CI	
Female (*ref*. Male)	**0.63**	**0.45–0.89**	**0.008**	0.98	0.73–1.30	0.869
Age (years)			0.612			**0.041**
1–14	Ref			Ref		
15–24	1.23	0.73–2.06		1.18	0.80–1.75	
25–34	0.99	0.59–1.64		1.45	0.98–2.14	
35–55	1.24	0.73–2.09		**1.74**	**1.16–2.62**	
Under observation ≤3 days (*ref*. OPD)	**6.01**	**3.33–10.84**	**< .001**	**4.32**	**2.33–8.02**	**< .001**
No YF vaccination* (*ref*. received vaccination)	**1.73**	**1.12–2.68**	**0.013**	**2.42**	**1.76–3.32**	**< .001**
Temperature at enrollment			**0.015**			0.752
Below 38.5°c	Ref			Ref		
≥ 38.5°c	**1.54**	**1.09–2.17**		1.05	0.77–1.44	
Fever duration prior to visit			0.081			0.087
1–2 days	Ref			Ref		
3 days	0.93	0.62–1.41		**1.40**	**1.04–1.89**	
4–7 days	1.53	0.97–2.43		1.25	0.87–1.80	
Presence of signs and symptoms (*ref*. absence)						
Rash	**2.59**	**1.46–4.59**	**0.001**	**1.54**	**1.00–2.37**	**0.049**
Retro-orbital pain	**7.37**	**3.69–14.71**	**< .001**	1.42	0.90–2.25	0.134
Nausea & vomiting	0.75	0.52–1.08	0.117	**1.36**	**1.01–1.82**	**0.042**
Cough	**0.36**	**0.21–0.63**	**< .001**	1.21	0.87–1.69	0.248
Loss of appetite	**0.46**	**0.30–0.71**	**< .001**	0.93	0.69–1.27	0.659
Headache	2.28	0.93–5.62	0.072	1.43	0.90–2.29	0.130
Constipation	1.08	0.23–4.97	0.926	0.52	0.24–1.10	0,087

*based on self-report

aOR = adjusted odds ratio

## Discussion

Recent reports of dengue outbreaks in Burkina Faso suggest substantial DENV transmission in this region. However, existing evidence on epidemiological characterization of dengue in Burkina Faso was limited in scope prior to this study. The current study collected population-based epidemiologic data in Ouagadougou during a 27-month period from 2014–2017, including all three months of the 2016 dengue outbreak. Our data demonstrated that dengue infection is an important cause of febrile illnesses, accounting for one-quarter of non-malarial febrile illness in patients seeking care at CSPSs in the study. This proportion was very high (55%) during the outbreak itself, but even outside the outbreak, a considerable proportion (15%) of non-malarial febrile episodes was dengue-positive. Since then, Ouagadougou has experienced another, larger, dengue outbreak in 2017 [16, 18]. Recent outbreaks and the current study indicate that DENV transmission is likely to be underestimated and underdiagnosed in Burkina Faso [16, 18].

Nonetheless, Burkina Faso is one of the countries in West Africa with better defined dengue virus transmission and burden. Several other countries with some existing data are Nigeria, Senegal, Ghana, and Sierra Leone. In Nigeria, presence of antibodies to DENV 2 was documented in 45% of 1816 human samples [30]. In Senegal, there were reported outbreaks of DENV 3 in 2009 with 196 individuals affected and 5 cases of dengue haemorragic fever (DHF) [31]. In Ghana, 3.2% among 218 children were found to show dengue IgM in 2014 [32]. In Sierra Leone, presence of antibody to all four serotypes of dengue virus was documented, based on neutralization test results on the samples from patients with fever of unknown origin [33]. While these reports suggest dengue presence in several West African countries, some with even high rates of infections, there is continued lack of data on dengue epidemiology in the region and highlighted need for improved surveillance system.

### Differences between outbreak and non-outbreak periods

The predominant DENV serotype identified from RT-PCR-positive outbreak cases in the study was DENV2 (Fig 4). This was consistent to the results of MoH/WHO investigation of the 2016 outbreak where DENV2 was the predominant serotype [16, 17]. DENV2 was also the dominant serotype detected in outbreaks in Burkina Faso in 1982 and 1983–1986 [9, 34]. The study found DENV3 to be predominant during the non-outbreak period preceding the 2016 outbreak. DENV3 was the dominant serotype in the 2013 outbreak in Burkina Faso [35]. A change in predominant DENV serotype may have fueled the outbreak in 2016. Although the current study did not determine DENV strain, DENV2 strains reported from ill French travelers returning from Burkina Faso in November 2016 were nearly identical to a DENV2 strain detected in Burkina Faso in 1983 [36]. This suggests that the 2016 outbreak may have been due to an endemic strain of DENV2 circulating in Burkina Faso for 30 years, perhaps maintained partly through a sylvatic cycle [36]. More detailed phylogenetic analysis of DENVs from the current study is planned.

Only a quarter of dengue-positive cases received a clinical diagnosis of suspected dengue in this study, with this proportion being only slightly higher during the 2016 outbreak (31% of dengue cases were suspected clinically) compared to outside the outbreak (17%). In the routine care system, clinicians in the CSPS refer to a guideline issued by the Burkina Faso MoH [37], primarily based on the 2009 WHO dengue guidelines. The dengue RDTs were made available at the CSPSs in the study, but the results of dengue RDT might not have contributed to the clinical assessment, if the results were not made available in time (dependent on patient volume and clinician availability). Dengue RDTs are typically unavailable for routine use in Africa; and many non-malaria febrile etiologies, including dengue, are likely to be under-diagnosed [12, 38]. Clinicians in Burkina Faso may need to consider dengue more frequently as a clinical diagnosis, with or without point-of-care assays.

Our multivariable analysis showed differing patterns of signs and symptoms associated with dengue-positivity during the outbreak period compared to non-outbreak periods. Rash was associated with dengue-positivity during both outbreak and non-outbreak periods. Rash is a common sign for dengue and listed in dengue classification in both 1997 and 2009 WHO dengue guidelines [3, 39, 40]. However, retro-orbital pain showed increased odds of dengue-positivity only during the outbreak. Retro-orbital pain, also listed in the 2009 WHO case definition, is another common sign associated with dengue-positivity [3, 39, 40]. Also, it was suggested that ocular symptoms, including retro-orbital pain, in dengue patients may possibly indicate thrombocytopenic state with increased likelihood of hemorrhage [41]. In our data, dengue-positive patients with retro-orbital pain were 5.8 times (95% C.I: 3.5–9.6, p < .001) more likely to require observation than dengue-positive patients without retro-orbital pain during the outbreak. During non-outbreak, it also showed a similar pattern with statistical significance, but with a wide confidence interval. Therefore, further information is needed for validation. While hemorrhagic signs were not commonly reported in our data, requiring observation may indicate severity of dengue illness and retro-orbital pain being associated with dengue-positive cases in the outbreak may indicate likely severity of dengue illness during the outbreak.

Our data showed a high proportion of individuals 15–40 years of age among dengue-positive cases in the outbreak period (a mean age of 26.8 years in dengue-positive patients). This was also found in the outbreak investigation by the Burkina Faso MoH with WHO where 70% of affected people were 25 years and older, with a mean age of 30 years [16]. It suggests that those in the labor force may be impacted, leading to significant economic and social burden [42]. Adjusted for age and gender, our model found higher odds that dengue-positive cases required observation, compared to non-dengue, during both outbreak (6.0 times) and non-outbreak (4.3 times) periods. Given the substantial proportion of dengue-positive cases among non-malarial febrile illnesses, this suggests that dengue may account for greater utilization of healthcare resources in CSPSs than other etiologies, during both outbreak and non-outbreak periods. As in many other parts of Africa, these primary healthcare centers have limited resources, such as beds [43], and could be especially overextended during outbreaks. Since the study only enrolled patients at CSPSs, the burden on the healthcare system due to dengue inpatients is unclear.

Self-report of not having received YF vaccination was associated with increased odds of dengue-positivity. A priori, one might have hypothesized cross-protection. However, the opposite phenomenon of a predisposition of YF vaccinated individuals to DHF has been suggested, with a possible explanation of cross-reactivity between antibodies from YF vaccination and dengue virus. [44]. Without much data on association of YF vaccination and dengue infection, self-reporting may be unreliable due to recall bias, and the study could not confirm YF vaccination using patient records.

### Study limitations

DENV transmission can vary substantially over time and space. Hence, the generalizability of the current study is limited by enrollment from the five selected CSPSs in the capital during the 27-month study period. We would have missed those community residents with relevant symptoms seeking care elsewhere than study centers, including private providers. In addition, patients with severe illness would have not been enrolled since they would likely have sought care directly at inpatient facilities; and subclinical and mild DENV infections would also not have been detected.

The study surveillance excluded patients with malaria RDT positive results, localizing signs or known/confirmed diagnosis with other diseases, possibly omitting co-infections of dengue with another pathogen. In particular, given the prevalence of malaria in this region, dengue and malaria co-infection were not included in this study and may require further investigation. Nevertheless, the available information on co-infections suggests they are uncommon [9, 45–48].

Performance of malaria RDTs, in terms of sensitivity, would depend on local conditions, especially the level of malaria transmission shown to be variable from reported incidence in Ouagadougou [49, 50]. There could have been misclassification among non-malarial patients (i.e. false negative results on malaria RDT included in the study being differently classified between dengue-positive and non-dengue groups). Also, this could vary by the level of dengue transmission (i.e. during and outside of the outbreak), leading to differential misclassification.

Our findings were based on outpatients and patients requiring observation, and clinical characteristics may be different for hospitalized patients and individuals with subclinical infections. Also, such findings may depend on other co-circulating pathogens endemic in the area, however our study did not confirm etiologies of non-dengue cases. Therefore, further information on the etiologies of non-dengue febrile cases may be needed to verify which signs are useful in distinguish non-dengue from dengue illnesses [51].

In our analysis, laboratory-confirmed and probable dengue cases were combined into the dengue-positive group. There may be some limitations with probable dengue being not as certain as lab-confirmed dengue. However, we performed analysis using 3 categories of dengue infection status (lab-confirmed-; probable-; and non-dengue) as part of sensitivity analysis and this yielded similar results (see S2–S4 Tables).

### Conclusion

Dengue is an important cause of non-malarial fever in Burkina Faso, both during and outside of outbreaks, despite being infrequently suspected by clinicians. Despite the many possible etiologies of febrile illness in this region, limited surveillance and diagnostic capacity will continue to pose challenges to dengue prevention and control. Additional longitudinal studies to better characterize dengue epidemiology and clinical presentation, including in inpatients and for subclinical/mild cases, along with encouraged use of dengue RDTs, would help to inform strategies to approach dengue countermeasures in this region.

## Supporting information

S1 Table Checklist. STROBE checklist.
(DOC)

S2 Table Demographic and clinical characteristics of patients by dengue infection status from the health facility-based fever surveillance established in Ouagadougou, Burkina Faso. (DOCX)

S3 Table Univariate logistic regression analyses showing significant indicators and their odds ratios between dengue-confirmed and non-dengue cases during the period of outbreak in the health facility-based fever surveillance. (DOCX)

S4 Table Univariate logistic regression analyses showing significant indicators and their odds ratios between dengue-confirmed and non-dengue cases during the period of non-outbreak in the health facility-based fever surveillance. (DOCX)
