# Supplementary material for: Clinical and epidemiologic characteristics associated with dengue during and outside the 2016 outbreak identified in health facility-based surveillance in Ouagadougou, Burkina Faso
Source: PLoS Negl Trop Dis. 2019 Dec 6;13(12):e0007882. doi: 10.1371/journal.pntd.0007882 (PMC6897397; doi:10.1371/journal.pntd.0007882)
Supplement: S1 Table — STROBE checklist. (DOC) [file pntd.0007882.s001.doc]

S1. STROBE Statement: Clinical and epidemiologic characteristics associated with dengue during and outside the 2016 outbreak identified in health-facility-based surveillance in Ouagadougou, Burkina Faso

|  | Item No | Recommendation |
| --- | --- | --- |
| **Title and abstract** | 1 | (*a*) Indicate the study’s design with a commonly used term in the title or the abstract (title on page 1) |
|  |  | (*b*) Provide in the abstract an informative and balanced summary of what was done and what was found (abstract on page 4) |
| Introduction | | |
| Background/rationale | 2 | Explain the scientific background and rationale for the investigation being reported (introduction, para 2-5) |
| Objectives | 3 | State specific objectives, including any prespecified hypotheses (introduction, para 5) |
| Methods | | |
| Study design | 4 | Present key elements of study design early in the paper (methods para 3, 4, and 5, Figure 2) |
| Setting | 5 | Describe the setting, locations, and relevant dates, including periods of recruitment, exposure, follow-up, and data collection (methods para 1-2, Figure 1, 3) |
| Participants | 6 | (*a*) *Cohort study*—Give the eligibility criteria, and the sources and methods of selection of participants. Describe methods of follow-up  *Case-control study*—Give the eligibility criteria, and the sources and methods of case ascertainment and control selection. Give the rationale for the choice of cases and controls  *Cross-sectional study*—Give the eligibility criteria, and the sources and methods of selection of participants (methods, para 3) |
|  |  | (*b*) *Cohort study*—For matched studies, give matching criteria and number of exposed and unexposed  *Case-control study*—For matched studies, give matching criteria and the number of controls per case |
| Variables | 7 | Clearly define all outcomes, exposures, predictors, potential confounders, and effect modifiers. Give diagnostic criteria, if applicable (methods, para 5-7) |
| Data sources/ measurement | 8 | For each variable of interest, give sources of data and details of methods of assessment (measurement). Describe comparability of assessment methods if there is more than one group (methods, para 7, 9) |
| Bias | 9 | Describe any efforts to address potential sources of bias (discussion para 4, 6) |
| Study size | 10 | Explain how the study size was arrived at (Figure 3) |
| Quantitative variables | 11 | Explain how quantitative variables were handled in the analyses. If applicable, describe which groupings were chosen and why (methods, para 5, 6) |
| Statistical methods | 12 | (*a*) Describe all statistical methods, including those used to control for confounding (methods, para 8-10) |
|  |  | (*b*) Describe any methods used to examine subgroups and interactions (methods, para 10, 12) |
|  |  | (*c*) Explain how missing data were addressed (results para 1) |
|  |  | (*d*) *Cohort study*—If applicable, explain how loss to follow-up was addressed  *Case-control study*—If applicable, explain how matching of cases and controls was addressed  *Cross-sectional study*—If applicable, describe analytical methods taking account of sampling strategy (Not applicable) |
|  |  | (*e*) Describe any sensitivity analyses (methods, para 10 - analysis just between dengue-confirmed and non-dengue groups, reported in tables S2-S4) |

| Results | | |
| --- | --- | --- |
| Participants | 13 | (a) Report numbers of individuals at each stage of study—eg numbers potentially eligible, examined for eligibility, confirmed eligible, included in the study, completing follow-up, and analysed (figure 3) |
|  |  | (b) Give reasons for non-participation at each (figure 3) |
|  |  | (c) Consider use of a flow diagram (figures 3) |
| Descriptive data | 14 | (a) Give characteristics of study participants (eg demographic, clinical, social) and information on exposures and potential confounders (results para 1-2, Tables 1 and 2) |
|  |  | (b) Indicate number of participants with missing data for each variable of interest (results para 1, explained on Fig 3 to have reached a sample size with complete information from variables used) |
|  |  | (c) *Cohort study*—Summarise follow-up time (eg, average and total amount) (Not applicable) |
| Outcome data | 15 | *Cohort study*—Report numbers of outcome events or summary measures over time |
|  |  | *Case-control study—*Report numbers in each exposure category, or summary measures of exposure |
|  |  | *Cross-sectional study—*Report numbers of outcome events or summary measures (Tables 1, 2, 3, 4) |
| Main results | 16 | (*a*) Give unadjusted estimates and, if applicable, confounder-adjusted estimates and their precision (eg, 95% confidence interval). Make clear which confounders were adjusted for and why they were included (Tables 3, 4, and 5) |
|  |  | (*b*) Report category boundaries when continuous variables were categorized (methods para 7) |
|  |  | (*c*) If relevant, consider translating estimates of relative risk into absolute risk for a meaningful time period (Not applicable) |
| Other analyses | 17 | Report other analyses done—eg analyses of subgroups and interactions, and sensitivity analyses (Table S2-S4) |
| Discussion | | |
| Key results | 18 | Summarise key results with reference to study objectives (discussion para 1) |
| Limitations | 19 | Discuss limitations of the study, taking into account sources of potential bias or imprecision. Discuss both direction and magnitude of any potential bias (discussion para 8-12) |
| Interpretation | 20 | Give a cautious overall interpretation of results considering objectives, limitations, multiplicity of analyses, results from similar studies, and other relevant evidence (discussion para 2-6) |
| Generalisability | 21 | Discuss the generalisability (external validity) of the study results (discussion para 8) |
| Other information | | |
| Funding | 22 | Give the source of funding and the role of the funders for the present study and, if applicable, for the original study on which the present article is based (financial disclosure on page 3) |

*Give information separately for cases and controls in case-control studies and, if applicable, for exposed and unexposed groups in cohort and cross-sectional studies.

**Note:** An Explanation and Elaboration article discusses each checklist item and gives methodological background and published examples of transparent reporting. The STROBE checklist is best used in conjunction with this article (freely available on the Web sites of PLoS Medicine at http://www.plosmedicine.org/, Annals of Internal Medicine at http://www.annals.org/, and Epidemiology at http://www.epidem.com/). Information on the STROBE Initiative is available at www.strobe-statement.org.
