# Supplementary material for: Clinical and epidemiologic characteristics associated with dengue during and outside the 2016 outbreak identified in health facility-based surveillance in Ouagadougou, Burkina Faso
Source: PLoS Negl Trop Dis. 2019 Dec 6;13(12):e0007882. doi: 10.1371/journal.pntd.0007882 (PMC6897397; doi:10.1371/journal.pntd.0007882)
Supplement: S2 Table — (DOCX) [file pntd.0007882.s002.docx]

S2. Table. Demographic and clinical characteristics of patients by dengue confirmation status from the health facility-based fever surveillance established in Ouagadougou, Burkina Faso

| Characteristics | Dengue-confirmed  (n=540) | Dengue-probable (n=200) | Non-dengue  (n=2189) | Total  (n=2929) | p-value |
| --- | --- | --- | --- | --- | --- |
| Age group (years) |  |  |  |  | **<.001** |
| 1-4 | 25 (4.6) | 12 (6.0) | 275 (12.6) | 312 (10.7) |  |
| 5-9 | 26 (4.8) | 17 (8.5) | 149 (6.8) | 192 (6.6) |  |
| 10-14 | 28 (5.2) | 17 (8.5) | 129 (5.9) | 174 (5.9) |  |
| 15-19 | 70 (13.0) | 15 (7.5) | 231 (10.6) | 316 (10.8) |  |
| 20-24 | 85 (15.7) | 25 (12.5) | 366 (16.7) | 476 (16.3) |  |
| 25-29 | 97 (18.0) | 37 (18.5) | 375 (17.1) | 509 (17.4) |  |
| 30-34 | 70 (13.0) | 24 (12.0) | 269 (12.3) | 363 (12.4) |  |
| 35-39 | 53 (9.8) | 18 (9.0) | 155 (7.1) | 226 (7.7) |  |
| 40-44 | 39 (7.2) | 18 (9.0) | 111 (5.1) | 168 (5.7) |  |
| 45-49 | 23 (4.3) | 10 (5.0) | 67 (3.1) | 100 (3.4) |  |
| 50-55 | 24 (4.4) | 7 (3.5) | 62 (2.8) | 93 (3.2) |  |
| Female | 333 (61.7) | 132 (66.0) | 1563 (71.4) | 2028 (69.2) | **<.001** |
| CSPS |  |  |  |  | **<.001** |
| Pazani | 81 (15.0) | 32 (16.0) | 400 (18.3) | 513 (17.5) |  |
| Zongo | 60 (11.1) | 31 (15.5) | 592 (27.0) | 683 (23.3) |  |
| CSPS 22 | 45 (8.3) | 20 (10.0) | 240 (11.0) | 305 (10.4) |  |
| CSPS25 | 206 (38.2) | 60 (30.0) | 502 (22.9) | 768 (26.2) |  |
| Juvenat Fille | 148 (27.4) | 57 (28.5) | 446 (20.4) | 651 (22.2) |  |
| Under observation ≤3 days/OPD | 117 (21.7)/423 (78.3) | 18 (9.0)/182 (91.0) | 45 (2.1)/2144 (97.9) | 180 (6.2)/2749 (93.9) | **<.001** |
| Mean days, fever duration prior to visit (SD) | 2.89 (1.20) | 3.03 (1.23) | 2.61 (1.22) | 2.69 (1.23) | **<.001** |
| Fever duration prior to visit |  |  |  |  | **<.001** |
| 1-2 days | 233 (43.2) | 68 (34.0) | 1153 (52.7) | 1454 (49.6) |  |
| 3 days | 162 (30.0) | 76 (38.0) | 634 (29.0) | 872 (29.85) |  |
| 4-7 days | 145 (26.9) | 56 (28.0) | 402 (18.4) | 603 (20.6) |  |
| Mean temperature at enrollment (SD) | 38.34 (0.75) | 38.14 (0.78) | 38.03 (0.78) | 38.09 (0.78) | **<.001** |
| Temperature at enrollment |  |  |  |  | **<.001** |
| Below 38.5°c | 333 (61.7) | 145 (72.5) | 1681 (76.8) | 2159 (73.7) |  |
| ≥ 38.5°c | 207 (38.3) | 55 (27.5) | 508 (23.2) | 770 (26.3) |  |
| Mean days, fever duration, entire illness (SD) | 4.77 (2.44) | 4.57 (2.71) | 4.04 (2.46) | 4.21 (2.49) | **<.001** |
| Prev. dengue infection | 10 (1.9) | 4 (2.0) | 2 (0.1) | 16 (0.6) | **<.001** |
| YF vaccination |  |  |  |  | **<.001** |
| Received | 83 (15.4) | 39 (19.5) | 824 (37.6) | 946 (32.3) |  |
| Not received | 457 (84.6) | 161 (80.5) | 1365 (62.4) | 1983 (67.7) |  |
| Clinical diagnosis |  |  |  |  |  |
| Suspected dengue | 144 (26.7) | 43 (21.5) | 12 (0.6) | 199 (6.8) | **<.001** |
| Undifferentiated fever | 379 (70.2) | 150 (75.0) | 1987 (90.8) | 2516 (85.9) |  |
| Non-dengue | 17 (3.2) | 7 (3.5) | 190 (8.7) | 214 (7.3) |  |
| URI (% of non-dengue) | 3 (17.6) | 2 (28.6) | 27 (14.2) | 32 (15.0) |  |
| Bronchitis | 2 (11.8) | 2 (28.6) | 30 (15.8) | 34 (15.9) |  |
| Pneumonia | 6 (35.3) | 0 | 21 (11.1) | 27 (12.6) |  |
| Viral syndrome | 1 (5.9) | 2 (28.6) | 11 (5.8) | 14 (6.5) |  |
| Diarrheal illness | 1 (5.9) | 1 (14.3) | 28 (14.7) | 30 (14.0) |  |
| Influenza | 1 (5.9) | 0 | 4 (2.1) | 5 (2.3) |  |
| Others | 3 (17.6) | 0 | 69 (36.3) | 72 (33.6) |  |
| Signs and symptoms (presence) |  |  |  |  |  |
| Rash | 73 (13.5) | 22 (11.0) | 163 (7.5) | 258 (8.8) | **<.001** |
| Fatigue | 446 (82.6) | 157 (78.5) | 1526 (69.7) | 2129 (72.7) | **<.001** |
| Headache | 518 (95.9) | 190 (95.0) | 1899 (86.8) | 2607 (89.0) | **<.001** |
| Retro-orbital pain | 103 (19.1) | 28 (14.0) | 107 (4.9) | 238 (8.1) | **<.001** |
| Neck pain | 8 (1.5) | 5 (2.5) | 47 (2.2) | 60 (2.1) | 0.556 |
| Nasal congestion | 16 (3.0) | 4 (2.0) | 105 (4.8) | 125 (4.3) | **0.044** |
| Rhinorrhea | 21 (3.9) | 9 (4.5) | 132 (6.0) | 162 (5.5) | 0.120 |
| Sore Throat | 7 (1.3) | 4 (2.0) | 64 (2.9) | 75 (2.6) | 0.088 |
| Cough | 62 (11.5) | 29 (14.5) | 354 (16.2) | 445 (15.2) | **0.024** |
| Sputum production | 2 (0.4) | 2 (1.0) | 30 (1.4) | 34 (1.2) | 0.123 |
| Nausea & vomiting | 190 (35.2) | 80 (40.0) | 635 (29.0) | 905 (30.9) | **<.001** |
| Diarrhea | 14 (2.6) | 9 (4.5) | 128 (5.9) | 151 (5.2) | **0.008** |
| Constipation | 6 (1.1) | 6 (3.0) | 85 (3.9) | 97 (3.3) | **0.005** |
| Abdominal pain | 195 (36.1) | 76 (38.0) | 639 (29.2) | 910 (31.1) | **<.001** |
| Nose bleeding | 6 (1.1) | 1 (0.5) | 10 (0.5) | 17 (0.6) | 0.198 |
| Gum bleeding | 4 (0.7) | 1 (0.5) | 2 (0.1) | 7 (0.2) | **0.021** |
| Loss of appetite | 251 (46.5) | 80 (40.0) | 739 (33.8) | 1070 (36.5) | **<.001** |
| Capillary refill >2 sec | 4 (0.7) | 4 (2.0) | 19 (0.9) | 27 (0.9) | 0.218 |
| Alterations to consciousness | 2 (0.4) | 4 (2.0) | 7 (0.3) | 13 (0.44) | **0.014** |
| Myalgia | 234 (43.3) | 85 (42.5) | 560 (25.6) | 879 (30.0) | **<.001** |
| Arthralgia | 315 (58.3) | 111 (55.5) | 953 (43.5) | 1379 (47.1) | **<.001** |
