# Supplementary material for: Clinical and epidemiologic characteristics associated with dengue during and outside the 2016 outbreak identified in health facility-based surveillance in Ouagadougou, Burkina Faso
Source: PLoS Negl Trop Dis. 2019 Dec 6;13(12):e0007882. doi: 10.1371/journal.pntd.0007882 (PMC6897397; doi:10.1371/journal.pntd.0007882)
Supplement: S3 Table — (DOCX) [file pntd.0007882.s003.docx]

S3 Table. Univariate logistic regression analyses showing significant indicators and their odds ratios of between dengue-confirmed and non-dengue cases during the period of outbreak in the health facility-based fever surveillance

| Characteristics | Total N | N (%) dengue confirmed  (n=357) | N (%)  Non- dengue  (n=349) | Univariate analysis  Dengue-confirmed vs. no dengue | | |
| --- | --- | --- | --- | --- | --- | --- |
|  |  |  |  | OR | 95% CI | p-Value |
| Age group (years) |  |  |  |  |  | 0.095 |
| 1-14 | 129 | 46 (35.7) | 66 (51.2) | Ref | - |  |
| 15-24 | 213 | 106 (49.8) | 92 (43.2) | **1.65** | **1.04-2.64** |  |
| 25-34 | 242 | 110 (45.5) | 114 (47.1) | 1.38 | 0.88-2.19 |  |
| 35-55 | 193 | 95 (49.2) | 77 (39.9) | **1.77** | **1.09-2.87** |  |
| Female* (*ref.* male) | 484 | 207 (42.8) | 237 (49.0) | **0.65** | **0.48-0.89** | **0.007** |
| Under observation** (*ref.* OPD) | 128 | 99 (77.3) | 18 (14.1) | **7.05** | **4.16-11.96** | **<.001** |
| Fever duration prior to visit* |  |  |  |  |  | **0.011** |
| 1-2 days | 330 | 147 (44.6) | 162 (49.1) | Ref | - |  |
| 3 days | 244 | 101 (41.4) | 115 (47.1) | 0.97 | 0.68–1.37 |  |
| 4-7 days | 203 | 109 (53.7) | 72 (35.5) | **1.67** | **1.15-2.42** |  |
| Temperature at enrollment * |  |  |  |  |  | **0.004** |
| Below 38.5°c | 468 | 195 (41.7) | 228 (48.7) | **Ref** | **-** |  |
| ≥ 38.5°c | 309 | 162 (52.4) | 121 (39.2) | **1.57** | **1.16-2.12** |  |
| No YF vaccination^A^* (*ref.* received vaccination) | 630 | 309 (49.1) | 267 (42.4) | **1.98** | **1.34-2.93** | **<.001** |
| Presence of signs and symptoms (*ref.* absence) |  |  |  |  |  |  |
| Rash* | 84 | 48 (57.1) | 24 (28.6) | **2.10** | **1.26–3.52** | **0.005** |
| Fatigue* | 620 | 300 (48.4) | 267 (43.1) | **1.62** | **1.11-2.35** | **0.012** |
| Retro-orbital pain** | 104 | 80 (76.9) | 12 (11.5) | **8.11** | **4.33-15.19** | **<.001** |
| Nasal congestion* | 21 | 5 (23.8) | 16 (76.2) | **0.30** | **0.11-0.82** | **0.019** |
| Rhinorrhea* | 28 | 6 (21.4) | 21 (75.0) | **0.27** | **0.11-0.67** | **0.005** |
| Cough** | 81 | 23 (28.4) | 53 (65.4) | **0.39** | **0.23-0.64** | **<.001** |
| Nausea & vomiting | 285 | 131 (46.0) | 131 (46.0) | 0.97 | 0.71-1.31 | 0.817 |
| Diarrhea | 21 | 5 (23.8) | 13 (61.9) | 0.37 | 0.13-1.04 | 0.060 |
| Abdominal pain | 263 | 127 (48.3) | 110 (41.8) | 1.20 | 0.88-1.64 | 0.255 |
| Loss of appetite* | 383 | 191 (49.9) | 166 (43.3) | 1.27 | 0.94-1.70 | 0.115 |
| Myalgia* | 366 | 189 (51.6) | 139 (38.0) | **1.70** | **1.26-2.29** | **<.001** |
| Arthralgia | 521 | 246 (47.2) | 226 (43.4) | 1.21 | 0.88-1.65 | 0.242 |
| Headache* | 749 | 350 (46.7) | 329 (43.9) | **3.04** | **1.27-7.28** | **0.013** |
| Sore throat | 11 | 3 (27.3) | 7 (63.6) | 0.41 | 0.11-1.62 | 0.204 |

Statistical significance of the frequencies: *p-value<0.05 **p-value<.001

^A^based on self-report
