# Supplementary material for: Clinical and epidemiologic characteristics associated with dengue during and outside the 2016 outbreak identified in health facility-based surveillance in Ouagadougou, Burkina Faso
Source: PLoS Negl Trop Dis. 2019 Dec 6;13(12):e0007882. doi: 10.1371/journal.pntd.0007882 (PMC6897397; doi:10.1371/journal.pntd.0007882)
Supplement: S4 Table — (DOCX) [file pntd.0007882.s004.docx]

S4 Table. Univariate logistic regression analyses showing significant indicators and their odds ratios between dengue-confirmed and non-dengue cases during the period of non-outbreak in the health facility-based fever surveillance

| Characteristics | Total N | N (%) dengue confirmed  (n=183) | N (%)  Non- dengue  (n=1840) | Univariate analysis  Dengue-confirmed vs. no dengue | | |
| --- | --- | --- | --- | --- | --- | --- |
|  |  |  |  | OR | 95% CI | p-Value |
| Age group (years)* |  |  |  |  |  | **<.001** |
| 1-14 | 549 | 33 (6.0) | 487 (88.7) | Ref | - |  |
| 15-24 | 579 | 49 (8.5) | 505 (87.2) | 1.43 | 0.91-2.27 |  |
| 25-34 | 630 | 57 (9.1) | 530 (84.1) | **1.59** | **1.02-2.48** |  |
| 35-55 | 394 | 44 (11.2) | 318 (80.7) | **2.04** | **1.27-3.28** |  |
| Female (*ref.* male) | 1544 | 126 (8.2) | 1326 (85.9) | 0.86 | 0.62-1.19 | 0.358 |
| Under observation** (ref. OPD) | 52 | 18 (34.6) | 27 (51.9) | **7.33** | **3.95-13.58** | **<.001** |
| Fever duration prior to visit* |  |  |  |  |  | 0.196 |
| 1-2 days | 1124 | 86 (7.7) | 991 (88.2) | Ref | - |  |
| 3 days | 628 | 61 (9.7) | 519 (82.6) | 1.35 | 0.96-1.91 |  |
| 4-7 days | 400 | 36 (9.0) | 330 (82.5) | 1.26 | 0.84-1.89 |  |
| Temperature at enrollment |  |  |  |  |  | 0.263 |
| Below 38.5°c | 1691 | 138 (8.2) | 1453 (85.9) | Ref | - |  |
| ≥ 38.5°c | 461 | 45 (9.8) | 387 (84.0) | 1.22 | 0.86-1.75 |  |
| No YF vaccination ^A^** (*ref.* received vaccination) | 1353 | 148 (10.9) | 1098 (81.2) | **2.86** | **1.95-4.18** | **<.001** |
| Presence of signs and symptoms (*ref.* absence) |  |  |  |  |  |  |
| Rash* | 174 | 25 (14.4) | 139 (79.9) | **1.94** | **1.23-3.06** | **0.005** |
| Fatigue** | 1509 | 146 (9.7) | 1259 (83.4) | **1.82** | **1.25-2.65** | **0.002** |
| Retro-orbital pain** | 134 | 23 (17.2) | 95 (70.9) | **2.64** | **1.63-4.28** | **<.001** |
| Nasal congestion | 104 | 11 (10.6) | 89 (85.6) | 1.26 | 0.66-2.40 | 0.485 |
| Rhinorrhea | 134 | 15 (11.2) | 111 (82.8) | 1.39 | 0.79-2.44 | 0.249 |
| Cough | 364 | 39 (10.7) | 301 (82.7) | 1.39 | 0.95-2.02 | 0.088 |
| Nausea & vomiting** | 620 | 59 (9.5) | 504 (81.3) | 1.26 | 0.91-1.75 | 0.163 |
| Diarrhea | 130 | 9 (6.9) | 115 (88.5) | 0.78 | 0.39-1.56 | 0.475 |
| Abdominal pain* | 647 | 68 (10.5) | 529 (81.8) | **1.47** | **1.07-2.01** | **0.018** |
| Loss of appetite* | 687 | 60 (8.7) | 573 (83.4) | 1.08 | 0.78-1.49 | 0.647 |
| Myalgia* | 513 | 45 (8.8) | 421 (82.1) | 1.10 | 0.77-1.57 | 0.601 |
| Arthralgia | 858 | 69 (8.0) | 727 (84.7) | 0.93 | 0.68-1.27 | 0.634 |
| Headache* | 1858 | 168 (9.0) | 1570 (84.5) | **1.93** | **1.12-3.32** | **0.018** |
| Sore throat | 64 | 4 (6.3) | 57 (89.1) | 0.70 | 0.25-1.95 | 0.495 |

Statistical significance of the frequencies: *p-value<0.05 **p-value<.001

^A^based on self-report
